# Spontaneous Rapid Resolution of Acute Epidural Hematoma in Childhood

**DOI:** 10.1155/2013/956849

**Published:** 2013-12-29

**Authors:** Ismail Gülşen, Hakan Ak, Enver Sösüncü, Alpaslan Yavuz, Nejmi Kiymaz

**Affiliations:** ^1^Yüzüncü Yıl University, School of Medicine, Van, Turkey; ^2^Bozok University, School of Medicine, Yozgat, Turkey; ^3^Akdeniz University, School of Medicine, Antalya, Turkey

## Abstract

Acute epidural hematoma is a critical emergency all around the world, and its aggressive diagnosis and treatment are of vital importance. Emergent surgical evacuation of the hematoma is known as standard management; however, conservative procedures are also used for small ones. Spontaneous rapid resolution of these hematomas has also been reported in eight pediatric cases. Various theories have been proposed to explain the underlying pathophysiology of this resolution. Herein, we are reporting a new pediatric case with spontaneously resolving acute epidural hematoma 12 hours after admission to the emergency room.

## 1. Introduction

Acute epidural hematoma is a serious complication of head injury; rapid diagnosis and early surgical evacuation of it are considered as the standard management [1, 2]. However, conservative followup also has made its place in the literature [1, 3, 4]. Since 1981, reported cases of spontaneous rapid resolution encourage the opinion of conservative followup [5]. Various theories have been proposed to explain the underlying mechanism of these cases [2, 5–7].

Herein, we are presenting an acute epidural hematoma case which resolved 12 hours after admitting to emergency department in a 4-year-old child and we are discussing the underlying mechanisms for our case in the light of the literature.

## 2. Case 

**Figure 1:**
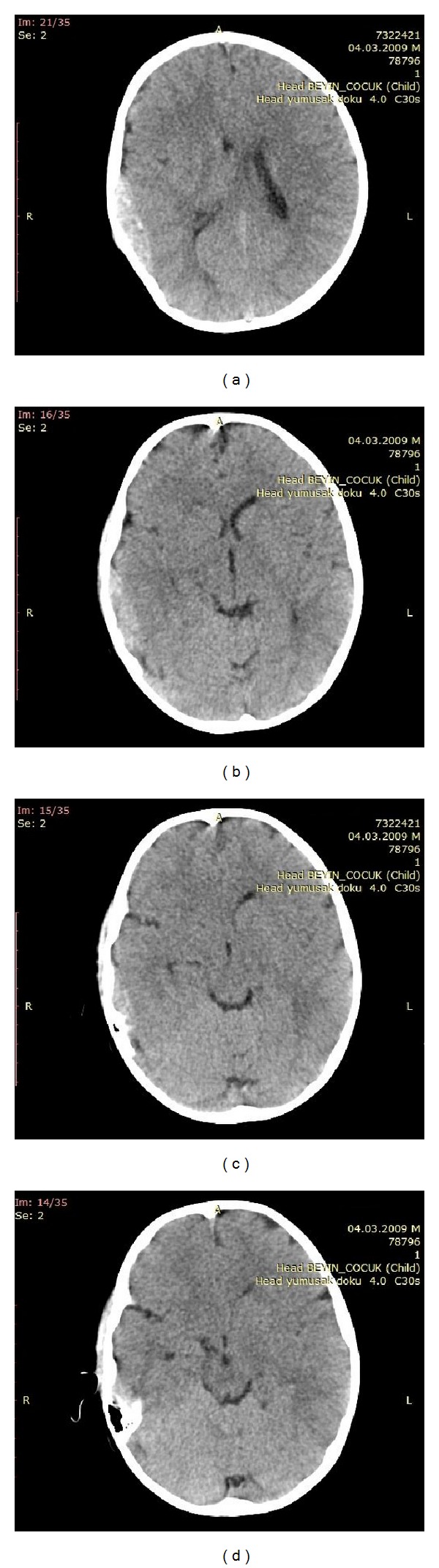
Axial CT image showing right sided epidural hematoma. (a) Inferior border of the hematoma at level of mastoid air cells. (b) Hematoma continuity at right temporal region. (c) Level of lateral ventricles. (d) Level of centrum semiovale.

**Figure 2:**
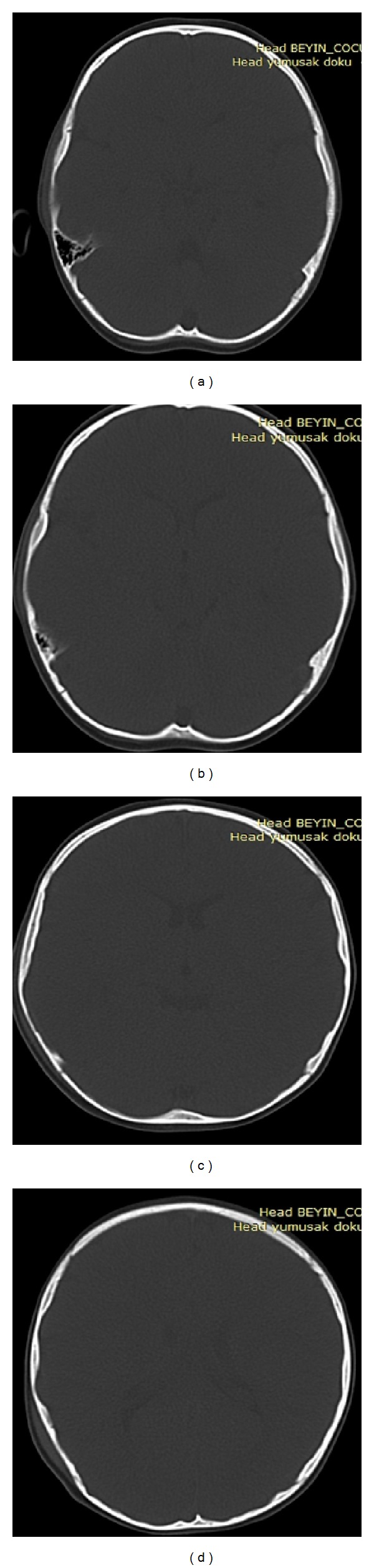
Axial CT image at different levels with no fracture. (a) Level of mastoid air cells. (b) Level of temporal region. (c) Level of lateral ventricles. (d) Level of centrum semiovale (there is a subgaleal hematoma indicated with white arrow).

Four-year-old boy was evaluated in the emergency department about one hour after a fall from a height of about two meters. His history was unremarkable except for mild headache. In the physical examination, there was edema, abrasion, and erythema on the right temporoparietal region. Neurological examination was normal. Computed tomography of the patient in the emergency department revealed acute epidural hematoma (Figure 1); maximal thickness was 17 mm, on the right temporoparietal region without bone fracture (Figure 2). Because of intact neurological examination, surgical evacuation was not considered. Patient was hospitalized for followup.

**Figure 3:**
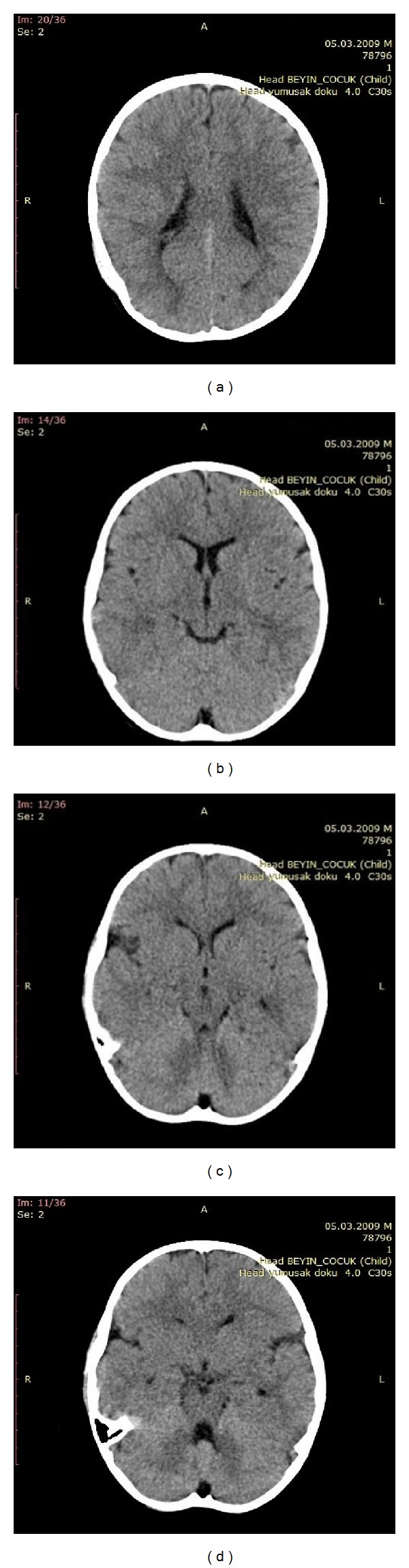
Axial CT image shows complete resolution of the hematoma. (a) Level of mastoid air cells. (b) Level of temporal region. (c) Level of lateral ventricles. (d) Level of centrum semiovale.

Twelve hours after hospitalization, we took computed tomography for controlling the size of the hematoma. We saw complete resolution of it (Figure 3). Patient was discharged after one day.

## 3. Discussion 

Although surgical evacuation is a settled opinion in the management of epidural hematomas, the opinion of conservative followup has made its ground in bonds in the literature. The main reasons of this may be summarized as increased presence of intensive care units more than before so the easy close followup of the patients with head injury, presence of neurosurgeon staff in many health centers during 24 hours, and easy access to computed tomography which is essential in the diagnosis and followup of such patients. Additionally, increased report of rapid spontaneous resolution of acute epidural hematoma cases after the first report in 1981 may also support the opinion of conservative followup [5].

Rapid spontaneous resolution of epidural hematoma has been reported in children as well as in adults. The literature research reveals 8 pediatric cases excluding our case [6]. However, there are 5 adult cases [7]. The oldest patient reported in the literature was 65 years old [8]. Various theories have been proposed to explain the underlying mechanisms of rapid spontaneous resolution. Some authors emphasized the existence of a potential communication with a fracture between intracranial and epicranial spaces [6, 9]. Increased intracranial pressure creates a pressure gradient between epidural hematoma and epicranial soft tissues so that hematoma is forced out of epidural space through fracture line. However, our patient did not have a fracture. Önal et al. also reported a case without fracture [10].

Resolution without fracture may be due to open cranial sutures. We consider that open sutures also cause a pressure gradient in a similar way as with fracture.

The second probable mechanism in spontaneous resolution is the pressure-induced redistribution secondary to brain swelling; however dissipation of hematoma seems harder due to tenacious adhesions between dura matter and skull [8, 11, 12]. Malek et al. proposed another theory that it may be due to an increased epicranial subgaleal interstitial pressure after injury, in which extracranial blood could leak to epidural space through a fracture due to a pressure gradient. When interstitial subgaleal pressure decreased, blood leaks back. This process takes about 18 hours [12]. Because of this reason, it seems that this theory is insufficient to explain the underlying mechanism for all cases especially for the cases that resolved in less than 18 hours. In our case, the time interval between falling and second CT image is about 13 hours. We do not have exact data about whether the volume of skull contents has an effect on resolution. According to our opinion, fully filling the skull with its contents (brain, cerebrospinal fluid, etc.) may be important in this resolution process. The presence of brain atrophy may block the rapid spontaneous resolution, because hematoma would have more epidural space than fully filling brain. The literature reveals only one case in advanced age (65 years old) [8].

When we look at the resolution times in pediatric cases, the longest time was 72 hours [6], and the shortest time was only one hour [10]. Bullock et al. reported that epidural hematoma less than 30 cm^3^ with less than 15 mm thickness and with less than 5 mm midline shift in patients with a Glasgow Coma Score greater than 8 without focal deficit could be managed nonoperatively with serial computed tomographic scanning and close observation in a neurosurgical center [13]. In our case thickness was 17 mm; however, total blood volume was less than 30 cm^3^, midline shift was less than 5 mm, and GCS was 15. We decided to manage the patient conservatively with serial CT. In our case, we detected the resolution after 12 hours. If we took the computed tomography sooner, perhaps we would see the resolution earlier. Önal et al. saw the resolution just after one hour, because of the deterioration of the general status of their patient. They retook CT and saw the resolution of acute epidural hematoma. Deterioration was due to hepatic artery injury in their patient [10]. We did not take computed CT earlier, because of the intact status of patient.

## 4. Conclusion 

The underlying pathophysiology of rapid spontaneous resolution of acute epidural hematomas has not yet been fully clarified, despite being more frequently reported cases, especially in children. We think that whether the skull contents fully fill the skull may be important in the pathophysiology of spontaneous rapid resolution. We believe that future experimental studies will shed light on our way.
